# Cybersickness Evaluation in Immersive Virtual Environments: A Systematic Review with Implications for Neurological Rehabilitation

**DOI:** 10.3390/jcm15010046

**Published:** 2025-12-21

**Authors:** María Fernández-Cañas, Rosa María Ortiz-Gutiérrez, Patricia Martín-Casas, Cecilia Estrada-Barranco, Selena Marcos-Antón, Aitor Blázquez-Fernández, Sofía Laguarta-Val, Carmen Jiménez-Antona, Roberto Cano-de-la-Cuerda

**Affiliations:** 1 International Doctorate School, Universidad Rey Juan Carlos, 28922 Alcorcón, Madrid, Spain; m.fernandezca.2019@alumnos.urjc.es (M.F.-C.); aitorblazquezfernandez@outlook.es (A.B.-F.); 2 Departamento de Fisioterapia, Universidad Complutense de Madrid, 28040 Madrid, Spain; rosaorti@ucm.es (R.M.O.-G.); pmcasas@ucm.es (P.M.-C.); 3 Departamento de Fisioterapia, Universidad Europea de Madrid, 28670 Villaviciosa de Odón, Madrid, Spain; 4 Departamento de Ciencias de la Salud, Universidad Villanueva, 28034 Madrid, Spain; 5 Asociación de Leganés de Esclerosis Múltiple, 28915 Leganés, Madrid, Spain; 6 Departamento de Fisioterapia, Terapia Ocupacional, Rehabilitación y Medicina Física, Universidad Rey Juan Carlos, 28922 Alcorcón, Madrid, Spain; carmen.jimenez@urjc.es (C.J.-A.);

**Keywords:** virtual reality, cybersickness, immersive technology, evaluation, assessment, neurorehabilitation, psychometric properties

## Abstract

**Introduction.** The proliferation of immersive virtual reality (VR) technologies has transformed the way individuals interact with digital environments, offering unprecedented opportunities in fields ranging from entertainment and education to healthcare and mental health interventions. Immersive VR is increasingly being implemented in motor and cognitive programs in neurorehabilitation, where patient safety and treatment adherence are critical. Despite its relevance, the conceptualization and measurement of cybersickness (CS) remain fragmented across disciplines, with various assessment tools developed in isolation, targeting different symptom domains, populations, and use contexts. **Aim.** The aim of this systematic review is to identify, categorize, and critically appraise all existing instruments and scales developed to measure CS associated with immersive VR use. The secondary objectives involve examining the psychometric properties of the identified instruments to provide robust evidence for clinicians in assessing CS associated with VR, thereby supporting future scale development and standardization. Additionally, a further objective is to evaluate the specific applicability of these instruments and scales for measuring CS within neurorehabilitation settings, given the growing use of immersive VR in clinical practice with neurological populations. **Methods.** This systematic review was conducted in accordance with the Guideline for reporting systematic reviews of outcome measurement instruments (PRISMACOSMIN). The review protocol was prospectively registered in the International Prospective Register of Systematic Reviews (PROSPERO). Data extraction was performed independently by the two reviewers, and discrepancies were resolved by consensus or consultation with a third reviewer. To assess the psychometric robustness of existing CS assessment instruments used in virtual reality settings, we applied the methodology proposed by the COSMIN (COnsensus-based Standards for the selection of health Measurement INstruments) initiative for systematic reviews of patient-reported outcome measures (PROMs). The evaluation was structured across three steps: Assessment of risk of bias; Quality of measurement properties and Summary of evidence; and Grading of Recommendations Assessment, Development and Evaluation (GRADE) guidelines. **Results.** Nine assessment instruments were included in this review. Following our analysis, the CSQ-VR and VRNQ received a Grade A recommendation, as they met sufficient criteria for both internal consistency and structural validity with high methodological quality. Few instruments have reported validations in specific clinical populations related to neurorehabilitation, including individuals with neurological conditions such as brain injury, multiple sclerosis (MS), migraine-associated vestibulopathy, vestibular disorders or cognitive impairment, indicating a critical gap in scale generalizability across clinical contexts. **Conclusions.** Despite the increasing use of immersive VR, few CS assessment tools have been designed and validated, reaching the CSQ-VR and VRNQ a Grade A recommendation. Among the nine identified assessment instruments, only the SSQ, MSAQ, CSQ-VR, VRNQ MSSQ and SEQ have been employed in samples with neurological disorders. There is a critical need for standardized CS VR-specific tools with robust psychometric properties to ensure safe and effective implementation in neurorehabilitation settings.

## 1. Introduction

Virtual reality (VR) is a simulation of a realistic or artificial environment created by a computer system, which allows the user to feel immersed in and interact with objects in that environment [1,2,3]. Depending on the “immersion” grade into the virtual environment experienced by the subject, VR devices can be divided into Non-Immersive VR tools, Semi-Immersive and Immersive ones. Immersive devices enable complete immersion by blocking visual perception of the real world, thereby restricting the user’s view to the virtual environment. This induces a strong sense of “being there”, commonly referred to as the “presence” effect [4,5].

The proliferation of these immersive VR technologies has transformed the way individuals interact with digital environments, offering unprecedented opportunities in fields ranging from entertainment and education to healthcare and mental health interventions [6,7]. Beyond these broad applications, VR has gained relevance in rehabilitation, where immersive environments support motor, cognitive, and sensory training. This growing adoption in clinical practice, and especially in neurorehabilitation, highlights the need to ensure patient safety and monitor potential adverse symptoms such as cybersickness (CS). Among the most pressing of these is the phenomenon often referred to as CS, encompassing symptoms such as disorientation, nausea, anxiety, sensory mismatch, and emotional unease during or after immersive VR exposure [8,9]. Weech S et al. (2018) [10] noted that CS is a multifactorial phenomenon. They highlighted four main domains: (a) Balance: Perceiving and controlling movement requires the integration of multisensory signals (vision, hearing, proprioception, and vestibular sense) to obtain accurate information about the body in space. Exposure to VR can generate a “sensory reorganization” in which the sensory relationships previously learned by the subject are modified. For example, delays in sensory feedback from different modalities can affect the perception, control, and performance of a given activity. Similarly, visual and vestibular signals regarding head position are not congruent during VR use. (b) Visual motion perception and vection: Individuals with greater sensitivity to three-dimensional visual motion are more likely to experience higher levels of discomfort. Those with higher visuospatial sensitivity may perceive greater visuo-vestibular mismatch. (c) Vestibular function: Clinical research in the field of vestibular dysfunction shows that subjects with alterations in vestibular labyrinth function do not present dizziness symptoms when exposed to rotating visual stimuli. Moreover, the vestibular system plays a fundamental role in postural control by detecting human fluctuations, so intersubject differences in vestibulo-inertial perception likely play a key role in symptom variability among individuals. (d) Additional factors: CS and different forms of motion sickness are closely linked to increased activation of the autonomic nervous system, with differences observed in the secretion of hormones such as vasopressin, adrenocorticotropic hormone, and growth hormone. The person’s sex may also play a determining role, as some studies report a higher prevalence of CS in women than in men, although other studies found no significant differences. These contradictory findings may be understood considering changes in hormonal activity and sympathetic nervous system function.

Head-mounted displays (HMDs) as wearable immersive tools require a dedicated area where the interactive environment is placed [11]. Due to the hardware’s cost-effectiveness, HMD-based immersive VR systems are presently the subject of intense research [12,13,14,15]. HMDs as rehabilitation tools have shown a positive effect on dexterity, gait performance, and dynamic balance in people with neurological disorders [16,17]. Specifically, HMD-based VR rehabilitation systems seem effective at increasing functionality [18].

Despite its relevance, the conceptualization and measurement of CS remain fragmented across disciplines, with various assessment tools developed in isolation, targeting different symptom domains, populations, and use contexts [9,19]. Given the increasing use of immersive VR in rehabilitation and the heightened vulnerability of neurological populations, it is essential to evaluate whether existing CS assessment instruments are appropriate for neurorehabilitation settings. This heterogeneity poses a significant challenge for comparative research, clinical evaluation, and the design of safer and more user-centered VR systems. One of the most comprehensive early attempts to address this issue was the systematic review by Davis et al. [9], which provided a detailed overview of CS in immersive virtual environments, including its symptoms, underlying theories, and contributing factors. While the authors acknowledged the importance of symptom evaluation and mentioned commonly used instruments such as the Simulator Sickness Questionnaire (SSQ), their focus was not on the systematic analysis or validation of these instruments. Nor did their review address how suitable these assessment instruments are across different user groups or clinical applications. Since then, the use of immersive VR has expanded rapidly, particularly in healthcare and rehabilitation. This raises important considerations about how CS is assessed, especially in vulnerable populations such as individuals with neurological disorders, who may experience increased sensitivity due to sensory or cognitive impairments [20]. Despite this, most measurement tools have been developed and tested primarily in healthy users [21].

To advance both scientific understanding and clinical application of immersive VR, it is essential that CS is assessed using valid, reliable, and appropriate instruments. Poorly defined measures refer to instruments in which key elements, such as symptom domains, scoring procedures, or interpretation rules, are not clearly described in the original publication. Inconsistently applied measures refer to instruments used differently across studies, for example, by varying the timing of administration, modifying scoring procedures (summed vs. averaged scores), or reporting only selected subscales. These issues became evident during data extraction when comparing how the same instruments were described or implemented across studies [22]. In clinical settings, inaccurate assessment instruments may overlook relevant symptoms or underestimate their impact, reducing patient safety and intervention success. Moreover, a clearer understanding of current measurement practices can inform the development of new instruments that are better suited to specific populations and use cases. This highlights the need for a systematic review that not only maps the existing assessment instruments currently in use but also evaluates their methodological quality and relevance across contexts. In response to this gap, and to establish a coherent framework for understanding and mitigating CS, it is essential to first identify, categorize, and critically evaluate the full spectrum of existing assessment instruments [19].

## 2. Aim

The aim of this systematic review is to identify, categorize, and critically appraise the available CS assessment instruments developed for immersive VR. The secondary objectives involve examining the psychometric properties of the identified instruments to provide robust evidence for clinicians in assessing CS associated with VR, thereby supporting future scale development and standardization. Additionally, a further objective is to evaluate the specific applicability of these assessment instruments for measuring CS within neurorehabilitation settings.

## 3. Methods

### 3.1. Search Strategy

This systematic review was conducted in accordance with the Guideline for reporting systematic reviews of outcome measurement instruments (PRISMA-COSMIN) (Supplementary Material Table S1) [23]. The review protocol was prospectively registered in the International Prospective Register of Systematic Reviews (PROSPERO) under registration number [CRD420251184492].

A comprehensive literature search was conducted by two reviewers (MFC and RCC) across these electronic databases: PubMed, Scopus, Web of Science, IEEE Xplore, Cochrane, PEDro and ACM Digital Library, covering all publications from database inception until 1 May 2025.

Combinations of keywords (Medical Subject Headings—MeSH—and free terms), including truncation for the different variations of words, were connected by Boolean operators as follows: “cybersickness”[Title/Abstract] OR “simulator sickness”[Title/Abstract] OR “virtual reality sickness”[Title/Abstract] OR “motion sickness”[Title/Abstract]) AND (“assessment scale”[Title/Abstract] OR “measurement”[Title/Abstract] OR “evaluation”[Title/Abstract] OR “questionnaire”[Title/Abstract] OR “instrument”[Title/Abstract]) AND (“virtual reality”[MeSH Terms] OR “VR”[Title/Abstract] OR “immersive environment”[Title/Abstract] OR “head-mounted display”[Title/Abstract]”

### 3.2. Study Selection

Studies were eligible if they met the following criteria: (i) population with no age limit; (ii) studies including the evaluation of CS after immersive VR treatment regardless of the study population; (iii) obtained by instrumental, numerical or observational procedures; (iv) analysis of at least one psychometric property of the measure; (v) studies written in English.

This systematic review excluded articles according to the following exclusion criteria: (i) studies published as study protocols, theoretical papers and clinical trials registration; (ii) studies that evaluate CS after no VR interventions (iii) systematic or no systematic reviews.

### 3.3. Data Collection

Data extraction was performed independently by the two reviewers using a standardized and piloted extraction form to collect data about: the scale name, purpose, domains assessed, number of items, interpretation of the total score, and usage context. When multiple studies validated the same scale, results were synthesized, and a narrative synthesis was conducted. Additionally, other characteristics of the assessment instruments such as author, target population, whether there are several versions of the scale (description of changes, if applicable), psychometric properties analyzed (i.e., validity, reliability or responsiveness), validation in clinical populations (if apply) and other observations were extracted. Due to the anticipated heterogeneity in instruments, study designs, and populations, a meta-analysis was not initially proposed. Instead, results were synthesized narratively and presented in tabular form.

All retrieved records were imported into a reference management software (EndNote, Clarivate Analytics, v.25.2), and duplicates were removed. Two independent reviewers (MFC and RCC) screened titles and abstracts against the eligibility criteria. Full texts of potentially relevant articles were obtained and assessed independently by the same reviewers. Inter-rater agreement was high, with only minor discrepancies, which were resolved by consensus or consultation with a third reviewer (SMA).

### 3.4. Evaluation of Selected Studies

To assess the psychometric robustness of existing CS assessment instruments used in VR settings, we applied the methodology proposed by the COSMIN (COnsensus-based Standards for the selection of health Measurement INstruments) initiative for systematic reviews of patient-reported outcome measures (PROMs) [24]. The evaluation was structured across three steps:

*Step 1: Assessment of risk of bias*

We evaluated the methodological quality of each included study using the COSMIN Risk of Bias checklist [25]. Each psychometric property assessed in a validation study (there are three domains covering various measurement properties: reliability -internal consistency, reliability, and measurement error-, validity -content validity, structural validity, hypothesis testing, cross-cultural validity, and criterion validity-, and responsiveness) was rated according to predefined criteria as “Very Good”, “Adequate”, “Doubtful”, or “Inadequate”, based on the quality of study design, statistical methods, and reporting [26].

The overall rating for the quality of each study for a measurement property was determined by the lowest rating for any standard: “the worst score counts” [27]. This step allowed for a transparent identification of potential limitations in the psychometric evidence of each instrument.

*Step 2: Quality of Psychometric Properties Measurement*

Measurement properties were extracted and evaluated according to the updated COSMIN criteria for assessing good measurement quality [26]. Each property was rated as: “+” (sufficient), “-” (insufficient), or “?” (indeterminate). This phase allowed us to assess the quality of each psychometric property analyzed.

*Step 3: Summary of evidence and recommendation Grade (GRADE)*

In accordance with COSMIN and GRADE (Grading of Recommendations Assessment, Development and Evaluation) guidelines [28], we synthesized the evidence per measurement property per scale, considering the number of studies, consistency of findings, quality (Risk of Bias), and precision. Each instrument was assigned an overall level of evidence (High, Moderate, Low, or Very Low), and a recommendation classification as: A (Recommended): sufficient evidence of adequate measurement properties; B (Potentially Recommended): promising but incomplete evidence; C (Not Recommended): evidence shows inadequate psychometric performance.

## 4. Results

### 4.1. Study Selection

**Figure 1:**
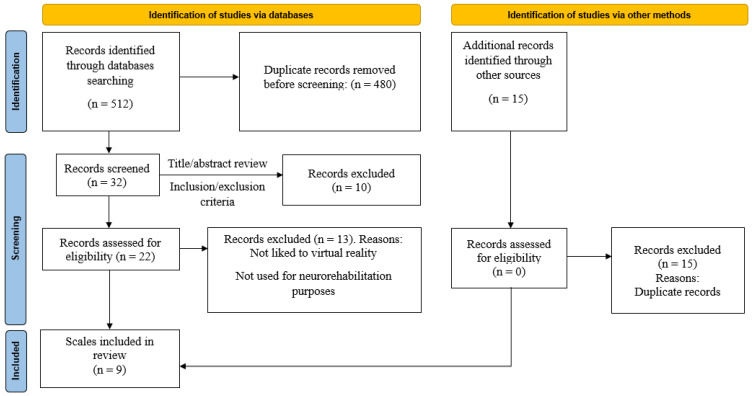
Flow diagram of the search strategy results.

The database search yielded 512 records, with 32 remaining after duplicate removal. Following title and abstract screening, 22 full-text articles were assessed for eligibility. Ultimately, nine assessment instruments were included in this review (see PRISMA flow diagram in Figure 1).

### 4.2. Characteristics of Included Assessment Instruments

Nine CS assessment instruments were included in this review. All of them were developed and/or validated between 1995 and 2023. The associated articles originated from a range of countries, specifically the USA (4), the UK (3), South Korea (1), Germany (1), and Spain (1). Table 1 summarizes the main characteristics of the identified CS scales.

The specifications of the scales analyzed are detailed below, including their structure, target population, domains assessed, scoring methods, and relevance to CS evaluation, particularly when applied in VR-based neurorehabilitation.

-The Simulator Sickness Questionnaire (SSQ) is one of the most widely used instruments for assessing simulator-induced discomfort, particularly in VR and other immersive environments. Developed by Kennedy et al. (1993) [19], the SSQ quantifies the severity and type of symptoms associated with simulator sickness, a condition closely related to motion sickness but induced by visual-vestibular discrepancies in simulated environments. The SSQ consists of 16 items that assess symptoms commonly experienced during or after simulation exposure. These symptoms are grouped into three subscales or domains: Nausea (N)—Includes symptoms such as increased salivation, stomach awareness, and burping. Oculomotor (O)—Includes visual discomfort symptoms such as eyestrain, blurred vision, and difficulty focusing. Disorientation (D)—Includes dizziness, vertigo, and perceptional instability. Each symptom is rated on a 4-point Likert scale ranging from “none” (0) to “severe” (3). The total SSQ score is derived by applying weighted sums to the symptom ratings in each subscale. The standard procedure includes computing the following: Nausea score = sum of relevant items × 9.54. Oculomotor score = sum of relevant items × 7.58. Disorientation score = sum of relevant items × 13.92. Total score = sum of all items × 3.74. Higher scores indicate greater severity of simulator sickness. While there is no universal cut-off, a total SSQ score above 20 is often interpreted as indicative of mild symptoms, and scores above 40 suggest moderate to severe simulator sickness.

The SSQ has been applied in several neurological populations and where it has demonstrated clinical utility for detecting VR-related symptoms. Nevertheless, formal psychometric validation studies in these groups remain limited, particularly regarding test–retest reliability and construct validity. Despite these limitations, the SSQ is the most widely used tool in immersive VR research.

-The Virtual Reality Sickness Questionnaire (VRSQ) is a self-report instrument developed to assess symptoms of CS specifically in VR environments. It was introduced by Kim et al. in 2018 [29] as a refined alternative to the SSQ, aiming to provide a more accurate measurement of VR-induced discomfort. The VRSQ comprises nine items divided into two subscales: four items assessing oculomotor symptoms (e.g., eyestrain and difficulty focusing) and five items assessing disorientation symptoms (e.g., dizziness and vertigo). Each item is rated on a 4-point Likert scale ranging from 0 (“not at all”) to 3 (“severe”). Subscale scores are obtained by summing the responses to the relevant items, and a total VRSQ score can be calculated by summing all item scores, providing an overall measure of VR-induced sickness, with higher scores indicating greater symptom severity. Most validation studies have been conducted with healthy participants [30].

-The Cybersickness Susceptibility Questionnaire (CSSQ) is a psychometric instrument designed to predict an individual’s likelihood of experiencing CS during VR exposure. Developed by Weech, Kenny, and Barnett-Cowan in 2019 [31], the CSSQ was created as a pre-exposure screening tool intended to estimate CS vulnerability based on personal history and baseline characteristics. The questionnaire comprises sixteen self-report items that assess a range of factors known to influence susceptibility, including previous experiences of motion sickness, migraine history, vestibular dysfunction, sensitivity to visual stimuli, and usage patterns of immersive technologies. Each item is rated using Likert-type response scales reflecting frequency or severity. A total susceptibility score is obtained by summing responses across all items, with higher scores indicating greater predicted vulnerability to VR-induced cybersickness. Although no universal cut-off score has been established, the CSSQ can assist in identifying individuals at comparatively higher risk before VR exposure. Current validation studies have been conducted primarily in healthy adult populations.

-The Motion Sickness Assessment Questionnaire (MSAQ) is a self-report instrument designed to evaluate the multidimensional nature of motion sickness symptoms. Developed by Gianaros et al. in 2001 [30], the MSAQ was created to overcome the limitations of earlier unidimensional scales by capturing a broader range of symptomatology associated with motion-induced discomfort. The questionnaire comprises sixteen items rated on a 9-point Likert scale ranging from 1 (“not at all”) to 9 (“severely”). These items are grouped into four symptom clusters: Gastrointestinal (e.g., nausea, queasiness, stomach discomfort), Central (e.g., dizziness, lightheadedness, disorientation), Peripheral (e.g., sweating, feeling hot or warm, clamminess), and Sopite-related (e.g., fatigue, drowsiness, irritability). Each item contributes to one domain score, allowing both domain-specific and total symptom severity assessments. Subscale scores are calculated by summing the items within each domain, and the total score—ranging from 16 to 144—is obtained by summing all item scores, with higher values indicating greater symptom severity. The multidimensional structure of the MSAQ provides a comprehensive overview of motion sickness profiles and supports the identification of individual differences. Validation studies have primarily been conducted with healthy participants and individuals with vestibular dysfunctions.

-The Fast Motion Sickness Scale (FMS), developed by Keshavarz and Hecht in 2011 [32], is a single-item verbal rating tool designed to assess the severity of motion sickness symptoms in real time during exposure to motion stimuli, including VR environments. The scale ranges from 0 (“no sickness at all”) to 20 (“frank sickness”), enabling rapid and continuous monitoring of symptom progression without interrupting the ongoing activity. Participants verbally report their current level of motion sickness at regular intervals (e.g., every minute) during exposure, allowing the scale to capture symptoms such as nausea, general discomfort, and stomach awareness while excluding unrelated sensations such as boredom or nervousness. Validation efforts to date have focused primarily on healthy populations.

-The Virtual Reality Neuroscience Questionnaire (VRNQ), developed by Kourtesis et al. (2019) [33], is a self-report instrument designed to assess both the quality of a VR software and the intensity of VR-induced symptoms and effects. The questionnaire evaluates four key domains: User Experience, assessing satisfaction and engagement with the VR environment; Game Mechanics, evaluating the intuitiveness, responsiveness, and functionality of system interactions; In-Game Assistance, measuring the clarity and helpfulness of instructions and prompts; and VR-Induced Symptoms and Effects, assessing symptoms such as nausea, dizziness, disorientation, fatigue, and instability during VR exposure. Each domain comprises five items, for a total of 20 items, rated on a 7-point Likert scale ranging from 1 (“not at all”) to 7 (“extremely”). Validation studies to date have been conducted primarily with healthy participants and individuals with cognitive impairments.

-The Cybersickness in Virtual Reality Questionnaire (CSQ-VR), developed by Kourtesis et al. (2023) [34], is a self-report instrument designed to assess the severity of CS symptoms experienced during VR exposure. It was created to address limitations found in existing measures such as the SSQ and the Virtual Reality Sickness Questionnaire (VRSQ). The CSQ-VR comprises six items divided into three subscales: Nausea, assessing symptoms such as stomach awareness and increased salivation; Disorientation, evaluating sensations such as dizziness and vertigo; and Oculomotor, measuring visual discomfort including eyestrain and blurred vision. Each subscale contains two items. Participants rate each item on a 7-point Likert scale ranging from 1 (“absent feeling”) to 7 (“extreme feeling”), and the total score is obtained by summing all item responses, with higher scores indicating greater severity of CS. Validation studies to date have been conducted with healthy participants as well as individuals with stroke and cognitive impairments.

-The Motion Sickness Susceptibility Questionnaire (MSSQ), developed by Golding in 1998 [35,36], is a widely used self-report instrument designed to assess an individual’s susceptibility to motion sickness across different contexts, including car, boat, airplane, and amusement rides. The questionnaire distinguishes between susceptibility during childhood and adulthood and consists of 18 items divided into two parts: the MSSQ-Short Childhood, which assesses motion sickness history up to age 12, and the MSSQ-Short Adulthood, which evaluates motion sickness experiences from age 12 onward. Responses are based on the reported frequency of symptoms during motion exposure and are rated using a Likert-type scale. Although initially validated in healthy populations, the MSSQ has been used in various clinical groups, including individuals with vestibular and neurological disorders, to help identify patients at higher risk of motion-related symptoms. A revisited version of the MSSQ evaluated its psychometric properties in modern VR contexts, particularly its utility in predicting VR-induced motion sickness. Additional studies have also examined the scale in populations such as individuals with vestibular migraine, MS, and stroke, where motion sickness and CS may be more pronounced due to altered sensory processing.

The Suitability Evaluation Questionnaire (SEQ) was designed specifically for VR systems [37] to evaluate user experience and perceived suitability in virtual environments. The questionnaire includes 14 questions, of which 13 are rated on a 5-point Likert scale, and one is a final yes/no question. Items 1–7 assess enjoyment, comfort and discomfort, feelings of success and control, realism, and the clarity of instructions. Items 8–11 focus on dizziness or nausea symptoms, eye discomfort, disorientation or confusion, and perceived progress in rehabilitation. Items 12 and 13 evaluate task difficulty and system usability. The global SEQ score ranges from 13 (poor suitability) to 65 (excellent suitability). The SEQ has demonstrated good internal consistency reliability.

### 4.3. Characteristics of Validation Studies Using Cybersickness Assessment Instruments Related to Immersive VR

To ease the comparison between the scales, a brief description of the initial validation population per scale, the VR technology employed, domains assessed and additional information about the validation (if apply) is shown in Table 2.

Regarding the population and technology employed, most instruments were initially validated in healthy populations, predominantly young adults, with a strong reliance on HMDs such as the Oculus Rift^®^ [37], HTC Vive^®^ [33,34] or Samsung Gear VR^®^ [29] (Table 2).

### 4.4. COSMIN-Based Evaluation of Measurement Properties

*Methodological Quality Assessment (Risk of Bias Assessment)* (Table 3 and Table 4): The methodological quality of the included studies was assessed using the COSMIN Risk of Bias checklist. Most studies evaluating structural validity, internal consistency, and reliability showed heterogeneous levels of methodological rigor. Table 3 indicates which psychometric properties were assessed for each instrument, while Table 4 presents the corresponding risk-of-bias ratings. Together, these tables make clear that several instruments lack complete reporting of key properties such as test–retest reliability, cross-cultural validity, and measurement error.

**Figure 2:**
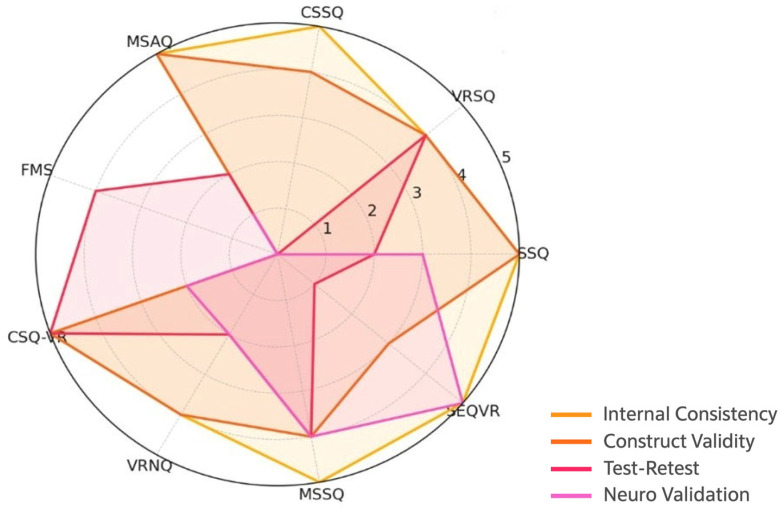
Radar chart comparing the psychometric properties (internal consistency, construct validity and test–retest reliability) across the nine cybersickness scales in immersive VR environments.

*Quality of Psychometric Properties Measurement* (Table 5, Figure 2): Each measurement property was evaluated using predefined criteria and classified accordingly:

Internal Consistency (+): Most scales demonstrated adequate internal consistency (Cronbach’s α > 0.70), especially SSQ, CSQ-VR, VRNQ, and MSSQ, justifying a positive rating. Table 5 provides an overall quality rating for each psychometric property across the included instruments, enabling a comparative interpretation of their methodological robustness and highlighting areas where evidence is limited or inconsistent. Figure 2 provides a visual comparison of the strength of each psychometric property across the nine instruments.

Structural Validity (++/+): Strong evidence (based on high-quality studies) indicates that structural validity is sufficient (+) for CSQ-VR and VRNQ; moderate evidence (+) for CSSQ and SEQVR; and insufficient (−) for PCA-only studies.

Test–Retest Reliability (+/− or ?): Sparse and inconsistent data were available. MSSQ and SSQ had partial reliability data (+/−), whereas for most scales this property was not assessed (?).

*Grading the Quality of Evidence (GRADE Approach)*: Using the GRADE framework, the overall quality of evidence per measurement property was determined by considering risk of bias, inconsistency, imprecision, and indirectness (Table 6). Grades follow COSMIN categories: A = recommended; B = potentially recommended; C = not recommended. Table 6 summarizes the GRADE-based overall recommendations for each instrument, integrating the quality, consistency, and completeness of the available psychometric evidence to support comparative interpretation and decision-making.

### 4.5. Clinical Application Contexts

Few scales have reported validations in specific clinical populations related to neurorehabilitation (SSQ, MSAQ, CSQ-VR, VRNQ MSSQ and SEQ), including individuals with neurological conditions such as brain injury, multiple sclerosis, migraine-associated vestibulopathy, vestibular disorders or cognitive impairment, indicating a critical gap in scale generalizability across clinical contexts (Table 2).

The reviewed CS assessment instruments vary in their application contexts, reflecting differences in their design and validation populations. The SSQ is the most widely used tool across both healthy and clinical samples, applicable in VR, flight simulators, and other immersive environments [19,38,39]. The SSQ, originally developed for flight simulators, the SSQ has been successfully adapted for VR environments and employed in patient groups including multiple sclerosis [40], individuals with vestibular disorders [41], and patients suffering from migraine-associated vestibulopathy [42,43]. The CSQ-VR and VRNQ are specifically designed for VR settings, primarily validated with healthy users but showing potential for future clinical applications such as stroke and cognitive impairment [33,44]. The MSSQ targets broader motion sickness assessment and susceptibility, respectively, with growing application in clinical settings [35,45]. The MSSQ, while primarily a predisposition measure rather than an immediate symptom scale, has been validated in populations with vestibular impairments, as well as the MSAQ scale [30], multiple sclerosis and stroke [35]. The SEQ focuses on VR-induced symptoms, with current use mostly in healthy participants and patients with stroke [29]. Lastly, the FMS offers a rapid, single-item assessment suited for quick symptom monitoring in VR or motion environments but lacks extensive clinical validation [32], as is also the case with the VRSQ and CSSQ scales.

Overall, while most scales are validated primarily in healthy individuals, a subset (notably the SSQ, MSAQ, CSQ-VR, VRNQ MSSQ and SEQ) have been validated in neurological populations, highlighting the need for further research to expand clinical validation across these tools.

Based on the current state of the art, a SWOT (Strengths, Weaknesses, Opportunities, and Threats) analysis has been developed to synthesize the key considerations regarding CS assessment scales and its possible development in the context of neurorehabilitation (Table 7).

## 5. Discussion

The aim of this review was to identify existing CS assessment instruments, examine their psychometric quality, and evaluate their applicability in neurorehabilitation. Our findings show that, despite the growing clinical relevance of immersive VR, validated tools specifically tested in neurological populations remain scarce.

Immersive VR is rapidly advancing as a valuable tool in rehabilitation and research, offering novel opportunities for motor, cognitive, and sensory recovery. However, CS—defined as a constellation of symptoms including nausea, dizziness, oculomotor disturbances, and disorientation—poses a significant barrier to patient adherence and safety in these interventions [7,8,46]. The accurate assessment of CS in clinical population, and specifically neurological patients, is therefore critical. Despite this clinical imperative, the number of validated CS assessment tools specifically tested in specific populations exposed to immersive VR remains strikingly limited.

### 5.1. Cybersickness Assessment Instruments and Psychometric Properties

The methodological quality indicated that most studies evaluating structural validity, internal consistency, and reliability showed heterogeneous levels of methodological rigor.

Very good ratings were assigned to structural validity assessments of the CSQ-VR and VRNQ, both of which used Exploratory Factor Analysis (EFA) with appropriate sample sizes, extraction methods, and justification of dimensionality. Adequate ratings were given to the CSSQ and SEQVR, where EFA was conducted but with limitations in sample representativeness or incomplete reporting. Doubtful or inadequate ratings were observed in studies using Principal Component Analysis (PCA) as a proxy for structural validity (SSQ, MSAQ, MSSQ), as well as in studies where key psychometric properties—such as test–retest reliability, hypothesis testing, or content validity—were insufficiently reported or methodologically unclear.

The evaluation of measurement properties revealed varying levels of psychometric robustness among the included instruments. Internal consistency was generally strong, with most scales, notably the SSQ, CSQ-VR, VRNQ, and MSSQ, demonstrating Cronbach’s α values above the acceptable threshold (>0.70), supporting their reliability in measuring consistent constructs. Structural validity was well-established for the CSQ-VR and VRNQ, supported by high-quality evidence, while moderate support was found for CSSQ and SEQVR. However, studies relying solely on principal component analysis (PCA) yielded insufficient evidence for structural validity. Test–retest reliability emerged as a notable limitation, with only partial or inconsistent data available for the MSSQ and SSQ, and a general lack of assessment across most tools. These findings underscore the need for more rigorous reliability testing, particularly over time, to strengthen the interpretability and generalizability of results derived from these instruments.

Using the GRADE framework, the overall quality of evidence per measurement property showed that the CSQ-VR and VRNQ received a Grade A recommendation, as they met sufficient criteria for both internal consistency and structural validity with high methodological quality. SSQ, MSSQ, and MSAQ were graded B, indicating acceptable but limited psychometric evidence. Finally, VRSQ, FMS, and CSSQ were assigned Grade C, either due to low quality of evidence or methodological weaknesses.

The SSQ [20] stands as the most extensively used instrument. Psychometric evaluations within these cohorts confirm the SSQ’s three-factor model (nausea, oculomotor, disorientation) with internal consistency coefficients ranging from 0.70 to 0.90, and test–retest reliability approximating 0.75 [33,39]. These properties underscore its robustness. On the other hand, the MSSQ has demonstrated good internal consistency (α = 0.80–0.94) and construct validity, correlating with observed susceptibility to VR-induced sickness [35]. However, the MSSQ’s use is largely prognostic and does not capture acute symptomatology during VR exposure. More recently developed instruments, such as the CSQ-VR, offer promising psychometric profiles tailored specifically for VR applications. The CSQ-VR exhibits excellent internal consistency (Cronbach’s α > 0.90) and a confirmed three-factor structure encompassing nausea, disorientation, and oculomotor symptoms, validated against physiological biomarkers including heart rate variability and galvanic skin response [34,44]. The VRSQ [29] and VRNQ [33] also demonstrate reliable factor structures and good internal consistency (α ~0.85–0.94) in healthy adults. Likewise, the FMS [31] provides an efficient single-item assessment tool with strong concurrent validity with the SSQ (r ~0.8) in healthy users. Other multidimensional instruments like the MSAQ [30] and the SEQ [37] have been designed for broader motion sickness or VR symptoms assessment with good to excellent internal consistency. However, their test–retest reliability has not been consistently reported or not reported.

### 5.2. Cybersickness Assessment Instruments and Validation Processes in Clinical Contexts Linked to Neurorehabilitation

Neurological disorders present heterogeneous symptom profiles that may influence both susceptibility and expression of CS during VR exposure. For instance, individuals with multiple sclerosis frequently experience vestibular dysfunction, fatigue, and sensory integration impairments, which have been linked to increased risk of VR-induced symptoms such as nausea, disorientation, and oculomotor disturbances. In a recent study by Pau et al. [41], 23 participants with multiple sclerosis (EDSS 1.5–5.5) were exposed to 10 min of immersive VR via HTC Vive^®^. The SSQ showed a significant increase in total score post-exposure (mean change +18.1; *p* < 0.001), with the most affected subscales being nausea and disorientation.

Similarly, individuals with stroke or Parkinson’s disease often exhibit cognitive impairment, altered sensorimotor control, and postural instability, which may further amplify susceptibility to CS. Tuena et al. [47] identified that more than 40% of stroke survivors reported mild to moderate symptoms of CS during VR-based rehabilitation protocols, especially when exposed to moving scenes or first-person perspectives. For patients with Parkinson’s disease, studies suggest that dopaminergic medications may interact with sensory processing and vestibular responses, potentially modulating the severity of CS symptoms during VR training [48]. Finally, the study of susceptibility to motion sickness linked to VR environments in vestibular patients (using the MSSQ) have been addressed [49,50].

The combined influence of cognitive decline, vestibular asymmetry, multisensory conflict, and pharmacologic effects necessitates tailored assessment tools validated specifically within neurological cohorts. However, despite their widespread use in healthy populations, instruments such as VRSQ, CSSQ or FMS have not yet been validated in clinical populations. To date, no current validation studies have conducted to examine CS in immersive VR among individuals with traumatic brain injury, Parkinson’s disease, or cerebral palsy. The absence of validated instruments in neurological populations may compromise the accurate detection of CS, limiting both patient safety and the interpretation of intervention outcomes. Clinically, this limitation means that cybersickness scores should be interpreted with caution when informing decisions on VR session progression, intensity, or discontinuation in neurological rehabilitation. This highlights the need for population-specific psychometric validation before these instruments are used in routine clinical practice.

### 5.3. Implications for Research and Clinical Practice

Future research should therefore prioritize the comprehensive validation of these VR-specific instruments in neurological populations. Essential psychometric properties warranting investigation include internal consistency, confirmatory factor analysis to verify domain structure, test–retest reliability to ensure temporal stability, and responsiveness to therapeutic changes during neurorehabilitation. Moreover, establishing normative data stratified by condition, disability severity, and demographic factors, along with minimal clinically important differences (MCIDs), will significantly enhance the interpretability and clinical utility of these tools.

The identified instruments differ in their conceptual scope. Some tools are designed to assess individual susceptibility or predisposition to cybersickness prior to VR exposure (e.g., MSSQ, CSSQ), whereas others focus on the measurement of acute symptoms experienced during or after VR sessions (e.g., SSQ, CSQ-VR, VRSQ, VRNQ). This distinction should guide instrument selection according to the intended application, such as pre-exposure screening, real-time monitoring, post-session symptom assessment, or outcome evaluation in clinical and research settings

In addition, heterogeneity across VR systems, including differences in HMD hardware, software design, and exposure duration, may influence the psychometric performance of cybersickness instruments. Such variability can modify symptom intensity and timing, which in turn may affect reliability estimates and limit score comparability across studies. Therefore, these technological differences should be taken into account when interpreting validation results.

Moreover, longitudinal designs examining CS symptom trajectories throughout VR therapy will inform optimal session duration, intensity, and patient safety protocols. The integration of objective physiological measures—such as heart rate variability, galvanic skin response, or postural sway—with subjective questionnaires could yield multidimensional assessment paradigms, improving symptom detection and management. Finally, when developing new hardware systems (HMDs) and custom virtual environments for immersive rehabilitative purposes, it is essential to assess their potential to induce CS. Evaluations should not only consider whether the system is immersive but also account for specific technological features such as the type of hardware, lens quality, virtual environment design, and exposure duration. These factors should be systematically evaluated using validated CS assessment scales.

This review highlights the critical need for the standardization and validation of CS assessment instruments to ensure their broad applicability and comparability across diverse populations. Additionally, there is an urgent requirement to develop and validate tools that are sensitive to the specific characteristics of various VR systems and user demographics, including clinical groups with neurological impairments. Furthermore, VR content developers should integrate validated CS measures within their design and testing protocols to enhance user safety, optimize experience, and facilitate regulatory compliance by ensuring adherence to emerging standards and guidelines for usability, accessibility, and adverse-event monitoring in immersive technologies. Addressing these priorities will be essential to advancing the effective and equitable implementation of immersive VR technologies in both research and clinical practice.

### 5.4. Limitations

This systematic review presents several methodological limitations. First, a meta-analytical synthesis of the collected data was not performed due to the heterogeneity in instruments, study designs, and populations. However, the main objective of this study was to deliver an updated qualitative synthesis of CS evaluation related to immersive VR devices. Moreover, a language restriction was applied, potentially limiting the comprehensiveness of the search and possibly omitting relevant validation studies published in languages other than English. In addition, the review may be affected by publication bias, as studies reporting positive or significant results are more likely to be published, while unpublished or null findings might not have been captured. Lastly, scarce psychometric data were found during data extraction for samples with neurological disorders. Findings and conclusions should be considered with appropriate caution given these constraints.

## 6. Conclusions

Despite the increasing use of immersive VR, few CS assessment tools have been designed and validated. The psychometric properties summarized in the COSMIN-based evaluation primarily reflect data derived from healthy populations. Following our analysis, the CSQ-VR and VRNQ received a Grade A recommendation, as they met sufficient criteria for both internal consistency and structural validity with high methodological quality. SSQ, MSSQ, and MSAQ were graded B, indicating acceptable but limited psychometric evidence. Finally, VRSQ, FMS, and CSSQ were assigned Grade C, either due to low quality of evidence or methodological weaknesses. Among the nine identified instruments, only the SSQ, MSAQ, CSQ-VR, VRNQ MSSQ and SEQ have been employed in samples with neurological disorders, so their comprehensive validation in neurorehabilitation contexts remains limited. There is a critical need for standardized CS VR-specific tools with robust psychometric properties to ensure safe and effective implementation in neurorehabilitation settings.

## Figures and Tables

**Table 1:** Main characteristics of the scales identified.

Scale Name and Authors	Country	Purpose	Domains Assessed	Number of Items	Estimated Administration Time *	Description of the Scale	Interpretation of Total Score	Usage Context
**Simulator Sickness Questionnaire (SSQ)** Kennedy et al., 1993 [19]	USA	Quantify simulator/cybersickness symptoms	3 subscales: Nausea, Oculomotor, Disorientation	16	5–7 min	Ordinal self-report scale. 4-point Likert scale	Symptoms are scored: 0 (none symptom) to 3 (severe symptom). Higher scores indicate greater severity of symptoms.	Widely used in simulator and VR research; applied across healthy subjects
**Virtual Reality Sickness Questionnaire (VRSQ)** Kim et al., 2018 [29,30]	Republic of Korea	Assess cybersickness in VR	2 subscales: Oculomotor Discomfort (4 items), Disorientation (5 items).	9	2–3 min	Rapid self-report 4-point Likert scale (from none to very severe)	Symptoms are scored: 0 (not at all) to 3 (severe) Higher scores indicate greater severity of symptoms.	Developed for VR-specific experiences; tested in healthy users
**Cybersickness Symptoms Questionnaire (CSSQ)** Del Cid et al., 2021 [31]	USA	Exploratory assessment of symptoms in VR	Oculomotor strain, Nausea, Dizziness, Fatigue	16	3–4 min	Estimated, Self-report questionnaire (recent validation) 4 level Likert scale (from none to severe)	Symptoms are scored: 0 (none) to 3 (severe) Higher scores indicating greater predicted vulnerability to VR-induced cybersickness.	Experimental use in VR-related studies, primarily with healthy users
**Motion Sickness Assessment Questionnaire (MSAQ)** Gianaros et al., 2001 [30]	USA	Evaluate multidimensional motion sickness	Items are grouped into four symptom clusters: Central, Peripheral, Gastrointestinal, Sopite-related symptoms	16	7–10 min	9 level Likert scale and multidimensional structure	Symptoms are scored: 0 (none symptom) to 9 (severe symptom) Higher total and subscale scores indicate increased severity of motion sickness	Used across transportation and VR settings; limited VR validation in healthy subjects
**Fast Motion Sickness Scale (FMS)** Keshavarz & Hecht, 2011 [32]	Germany	Rapid screening for motion sickness severity	General discomfort (single item, repeated over time)	1	5 min	Estimated, short, and unidimensional self-report scale.	Single question where 0 (no symptom) to 20 (clear/severe symptom).	High temporal resolution; useful for real-time monitoring in VR. Healthy subjects.
**Virtual Reality Neuroscience Questionnaire (VRNQ)** Kourtesis et al., 2021 [33]	UK	Assess adverse symptoms and content quality in VR sessions	4 domains: User experience, Game Mechanics, In-Game Assistance and VR-Induced Symptoms and Effects. Each domain comprises 5 items	20	6–7 min	Self-report scale 7-point Likert scale	Symptoms are scored: 1 (not at all) to 7 (extremely). Higher scores indicate greater severity symptoms.	Evaluates both symptoms and technical design, for safe VR exposure
**Cybersickness in Virtual Reality Questionnaire (CSQ-VR)** Kourtesis et al., 2023 [34]	UK	Comprehensive measure for VR-related cybersickness	3 subscales: Oculomotor, Nausea and Disorientation. Each subscale contains 2 items.	6	4–5 min	Self-report scale 7-point Likert scale 6 items divided into 3 subscales.	Symptoms are scored: 1 (absent feeling) to 7 (extreme feeling). Total score is obtained by summing all items responses. Higher scores indicate greater severity symptoms.	Designed for HMD-based VR; validated against SSQ and VRSQ. Healthy subjects.
**Motion Sickness Susceptibility Questionnaire (MSSQ-Short)** Golding, 1998; Won et al., 2022 [35,36]	UK (Golding); USA (Won)	Predict individual vulnerability to motion sickness	Past experiences in various transport modes, divided into 2 parts: Short Childhood, and Short Adulthood.	18 (items defined by Golding); 10 (items defined by Won)	8–10 min	Self-report and retrospective scale 4-point Likert scale	Symptoms are scored: 0 (never) to 3 (frequently). Higher scores reflect greater historical susceptibility	Pre-VR screening tool; validated for general and VR contexts
**Suitability Evaluation Questionnaire for VR (SEQ)** Gil-Gómez et al., 2013 [37]	Spain	Evaluate usability and suitability of VR systems	User experience and perceived suitability in virtual environments.	14	3–5 min	Self-report scale. 5-point Likert scale. (the first 13 questions) The last one is a final yes/no question.	Score ranges: from 13 (poor suitability) to 65 (excellent suitability.	Developed for rehabilitation settings, including postural VR therapy

* Notes about administration times: Times may vary slightly depending on the cognitive profile of the users, especially in neurological populations. Shorter scales such as the FMS and VRSQ are useful in clinical settings where rapid assessment is required. Scales such as CSQ-VR or SSQ may take longer because of their larger number of items or greater complexity of wording.

**Table 2:** Characteristics of Validation Studies Related to Immersive VR.

Scale Name	Initial Validation Population	VR System/HMD Used	Domains Assessed	Internal Consistency	Construct Validity	Test–Retest Reliability	Additional Information About Validation in Specific Populations Related to Neurorehabilitation *
**SSQ** [19]	Military, aviation personnel	Flight Simulators (non-VR)	Nausea, Oculomotor, Disorientation	α ** = 0.70 to 0.90	3-factor (Oculomotor, Nausea, Disorientation)	ICC *** = 0.75	Multiple sclerosis, Vestibular migraine, stroke
**VRSQ** [29,30]	Healthy students	Oculus Rift CV1	Oculomotor, Disorientation	α = 0.87	2-factor (Oculomotor, Disorientation)	ICC = 0.79	None
**CSSQ** [31]	Healthy adults	Immersive VR (unspecified)	Oculomotor, Nausea, Dizziness, Fatigue	α = 0.91	Exploratory (2 factors: nausea, visual)	Not reported	None
**MSAQ** [30]	Healthy adults	Physical simulators	GI, Central, Peripheral, Sopite	α = 0.88–0.93	4-factor (GI, Central, Peripheral, Sopite)	Not consistently reported	Vestibular disorders (reported use)
**FMS** [32]	Healthy volunteers	Various HMDs/screens	General discomfort (1 item)	Not applicable	Not applicable	High (reported)	None
**VRNQ** [33]	Healthy adults	HTC Vive Pro Eye	Cybersymptoms, UX, Visual and Interaction fidelity	α = 0.86–0.94	4-factor (Symptoms + UX)	Not reported	Pilot study in mild cognitive impairment
**CSQ-VR** [34]	Healthy adults	HTC Vive Pro Eye	Oculomotor, Nausea, Disorientation, Headache, Fatigue	α = 0.95	2-factor confirmed; convergent with SSQ	ICC = 0.93	Pilot study in stroke, cognitive impairment
**MSSQ** [35,36]	General population	Oculus Rift S	Motion history (childhood and adult)	α = 0.80–0.94	2-form: childhood/adult	ICC > 0.80	Vestibular disorders, multiple sclerosis and stroke
**SEQ** [37]	Patients with balance disorders	Custom screen-based VR	Safety, Comfort, Satisfaction, Movement fidelity	α = 0.92	Content and face validity	Not reported	Stroke

* Additional notes: SSQ and MSSQ are the most widely used but not developed for immersive VR; adapted post hoc. CSQ-VR and VRNQ are purpose-built for VR. SEQ is the only scale initially validated in neurological patients, though not a symptom scale per se. ** α: Cronbach’s alpha. *** ICC: Intraclass Correlation Index.

**Table 3:** Psychometric Properties Assessed per Scale.

Scale	PROM Development	Content Validity	Structural Validity	Internal Consistency	Cross-Cultural Validity	Reliability	Measurement Error	Criterion Validity	Construct Validity (Hypothesis Testing)	Responsiveness
**SSQ** [19]	Expert-derived items from motion sickness scales	No formal content validity study	EFA * + CFA	α **	Not tested	ICC (in some adaptations)	Not tested	Not applicable	Correlation with simulator exposure	Not tested
**VRSQ** [29,30]	Item generation by experts	Content validity by panel review	EFA	α	Not tested	Not tested	Not tested	Not tested	Comparison across device types	Not tested
**CSSQ** [31]	Developed via literature + expert input	Pilot-tested for clarity	CFA	α	Not tested	SEM ***, ICC	SEM	Not tested	VR vs. non-VR task discomfort	Not tested
**MSAQ** [30]	Derived from motion sickness framework	Face/content validity via expert consensus	EFA	α	Not tested	ICC (subsample)	Not tested	Not tested	Correlations with nausea scales	Not tested
**FMS** [32]	Short form via item reduction	No specific content validity study	EFA	α	Not tested	Not tested	Not tested	Not tested	Comparison to other measures	Not tested
**VRNQ** [33]	Developed via iterative item testing	Face validity + user feedback	CFA	α	Not tested	Not tested	Not tested	Not tested	Comparison across domains	Not tested
**CSQ-VR** [34]	Developed from previous VR instruments	Cognitive debriefing	CFA	α	Not tested	Not tested	Not tested	Not tested	Moderate correlations with usability	Not tested
**MSSQ** [35,36]	Developed from motion exposure theory	Face/content via large sample feedback	PCA	α	Tested (translations)	ICC	SEM	Not tested	Correlation with motion exposure	Not tested
**SEQ** [37]	Expert-developed items for VR rehab	No formal study	Not reported	α	Not tested	Not tested	Not tested	Not tested	Preliminary group comparisons	Not tested

* EFA: Exploratory Factor Analysis; CFA: Confirmatory Factor Analysis; PCA: Principal Component Analysis. ** α: Cronbach’s alpha. *** SEM: Standard Error of Measurement.

**Table 4:** Psychometric Properties Assessed and Risk of Bias per Scale.

Scale	PROM Development	Content Validity	Structural Validity	Internal Consistency	Cross-Cultural Validity	Reliability	Measurement Error	Criterion Validity	Construct Validity (Hypothesis Testing)	Responsiveness
**SSQ** [19]	Adequate	— *	Adequate	Adequate	—	Doubtful	—	—	Adequate	—
**VRSQ** [29,30]	Adequate	Adequate	Adequate	Adequate	—	—	—	—	Doubtful	—
**CSSQ** [31]	Adequate	Adequate	Very Good	Very Good	—	Adequate	Adequate	—	Adequate	—
**MSAQ** [30]	Adequate	Adequate	Adequate	Adequate	—	Adequate	—	—	Adequate	—
**FMS** [32]	Doubtful	—	Adequate	Adequate	—	—	—	—	Doubtful	—
**VRNQ** [33]	Adequate	Adequate	Very Good	Very Good	—	—	—	—	Adequate	—
**CSQ-VR** [34]	Adequate	Adequate	Very Good	Very Good	—	—	—	—	Adequate	—
**MSSQ** [35,36]	Very Good	Adequate	Adequate	Very Good	Very Good	Adequate	Adequate	—	Very Good	—
**SEQ** [37]	Doubtful	—	Inadequate	Doubtful	—	—	—	—	Doubtful	—

* —: no data.

**Table 5:** Overall Quality Rating for Each Psychometric Property.

Scale	PROM Development	Content Validity	Structural Validity	Internal Consistency	Cross-Cultural Validity	Reliability	Measurement Error	Criterion Validity	Construct Validity (Hypothesis Testing)	Responsiveness
**SSQ** [19]	+ *	**−**	**+**	**+**	**−**	**+/−**	**−**	**−**	**+**	**−**
**VRSQ** [29,30]	**+**	**+**	**+**	**+**	**−**	**−**	**−**	**−**	**+/−**	**−**
**CSSQ** [31]	**+**	**+**	**++**	**++**	**−**	**+**	**+**	**−**	**+**	**−**
**MSAQ** [30]	**+**	**+**	**+**	**+**	**−**	**+**	**−**	**−**	**+**	**−**
**FMS** [32]	**+/−**	**−**	**+**	**+**	**−**	**−**	**−**	**−**	**+/−**	**−**
**VRNQ** [33]	**+**	**+**	**++**	**++**	**−**	**−**	**−**	**−**	**+**	**−**
**CSQ-VR** [34]	**+**	**+**	**++**	**++**	**−**	**−**	**−**	**−**	**+**	**−**
**MSSQ** [35,36]	**++**	**+**	**+**	**++**	**++**	**+**	**+**	**−**	**++**	**−**
**SEQ** [37]	**+/−**	**−**	**−**	**+/−**	**−**	**−**	**−**	**−**	**+/−**	**−**

* Psychometric properties: ++ → Very good or excellent quality for that property. + → Good or acceptable quality. +/− → Moderate quality, acceptable but with some limitations. − → Low or poor quality for that property.

**Table 6:** GRADE Recommendation Table for Cybersickness Scales.

Scale	GRADE Level	Recommendation	Justification
**CSSQ** [31]	**A**	Recommended	High-quality evidence for internal consistency and structural validity. Developed using COSMIN standards. Recent and VR-specific.
**CSQ-VR** [34]	**A**	Recommended	Demonstrates strong psychometric properties (++ in key areas), specific to VR, developed with rigorous methods.
**VRNQ** [33]	**A**	Recommended	Covers multiple domains of VR experience including cybersickness; robust validation, strong internal consistency and structure.
**MSSQ** [35,36]	**A**	Recommended with scope	Strong psychometric performance, but not originally designed for VR; retrospective nature may limit utility in acute settings.
**SSQ** [19]	**B**	Acceptable with caution	Widely used and validated, but limited recent structural validation, and not VR-specific. Mixed test–retest reliability evidence.
**MSAQ** [30]	**B**	Acceptable	Developed for motion environments; adequate psychometric data, but not VR-specific. Suitable for comparison studies.
**VRSQ** [29,30]	**B**	Acceptable	Brief, validated for VR, good internal consistency; lacks test–retest data and structural validity across populations.
**FMS** [32]	**C**	Not recommended without complementary measures	Sparse evidence, limited construct validation, no data on reliability or factor structure.
**SEQ** [37]	**C**	Not recommended	Limited psychometric evaluation, negative structural validity findings, lacks evidence in multiple properties.

**Table 7:** SWOT Analysis of Cybersickness Assessment Scales in Neurorehabilitation.

**Strengths**	**Weaknesses**
Several instruments demonstrate acceptable internal consistency and construct validity within clinical or semi-clinical contexts. Established tols like the SSQ and MSAQ offer historical continuity and inter-study comparability. The availability of both brief (VRSQ, FMS, SEQVR) and multidimensional (MSAQ, VRNQ) formats provide flexibility for different clinical and research applications.	Most scales have limited or no validation in neurological populations, despite their frequent application in neurorehabilitation. A general lack of test–retest reliability data and sensitivity to clinical change undermines longitudinal applicability. Heterogeneity in the VR systems or HMDs used during scale validation limits generalizability across technological setups. Several tools lack comprehensive psychometric evaluation or peer-reviewed validation studies.
**Opportunities**	**Threats**
Development of VR-specific tools tailored for neurological disorders such as Parkinson’s disease, vestibular dysfunction, or post-stroke impairments. Integration with clinical metrics (e.g., balance, cognitive load, autonomic markers) to enhance ecological and clinical validity. Utilization of objective physiological or behavioral data (e.g., eye tracking, postural sway) to support and triangulate self-report measures. Potential to define clinically meaningful cut-off points and severity thresholds for cybersickness in immersive rehabilitation.	Rapid technological evolution in VR hardware (e.g., resolution, latency, optics) may render some existing scales outdated. Increasing use of custom-built VR environments without standardized CS evaluation may jeopardize patient safety Risk of false negatives or symptom underestimation when using non-validated scales in clinical neurological settings. Absence of international consensus on standardized tools limits harmonization across studies and clinical practice.

## Data Availability

The data are available from the corresponding authors upon reasonable request.

## Supplementary Materials

The following supporting information can be downloaded at: https://www.mdpi.com/article/10.3390/jcm15010046/s1.
