# Supplementary material for: Cybersickness Evaluation in Immersive Virtual Environments: A Systematic Review with Implications for Neurological Rehabilitation
Source: J Clin Med. 2025 Dec 21;15(1):46. doi: 10.3390/jcm15010046 (PMC12787253; doi:10.3390/jcm15010046)
Supplement: Supplementary file 1 [file jcm-15-00046-s001.zip › jcm-4031321-supplementary.pdf]

PRISMA - COSMIN OUTCOME Measurement Instruments

Full reports

Section and Topic	#	Checklist item[a]	Location
TITLE			
Title	1	Identify the report as a systematic review and include as applicable the following (in any order): outcome domain of interest, population of interest, name/type of OMIs of interest, and measurement properties of interest.	Title and abstract, page 1 - 2
ABSTRACT			
OPEN SCIENCE			
Funding[b]	2.2	Specify the primary source of funding for the review.	Funding, page 23
Registration	2.3	Provide the register name and registration number.	Methods, search strategy, page 6 (paragraph 1)
BACKGROUND			
Objectives	2.4	Provide an explicit statement of the main objective(s) or question(s) the review addresses.	Introduction, aim, page 5 (paragraph 2)
METHODS			
Eligibility criteria	2.5	Specify the inclusion and exclusion criteria for the review.	Methods, study selection, page 6 (paragraphs 4, 5)
Information sources	2.6	Specify the information sources (e.g., databases, registers) used to identify studies and the date when each was last searched.	Methods, search strategy, page 6 (paragraph 2, 3)

Section and Topic	#	Checklist item[a]	Location
Risk of bias	2.7	Specify the methods used to assess risk of bias in the included studies.	Methods, Evaluation of selected studies, step 1: assessment of risk of bias, page 8 (paragraph 1, 2)
Measurement properties	2.8	Specify the methods used to rate the results of a measurement property.	N/A
Synthesis methods	2.9	Specify the methods used to present and synthesize results.	Methods, Data collection, page 7 (paragraph 1)
RESULTS			
Included studies	2.10	Give the total number of included OMIs and study reports.	Results, Study selection, page 9 (paragraph 2) and Characteristics of included assessment instruments, page 9 (paragraph 3)
Synthesis of results	2.11	Present the syntheses of results of OMIs, indicating the certainty of the evidence.	Results, COSMIN -

Section and Topic	#	Checklist item[a]	Location
			based evaluation of measurement properties, table 6
DISCUSSION			
Limitations of evidence	2.12	Provide a brief summary of the limitations of the evidence included in the review (e.g., study risk of bias, inconsistency, and imprecision).	Results, COSMIN-based evaluation of measurement properties, page 15, (Table 4)
Interpretation	2.13	Provide a general interpretation of the results and important implications.	Discussion, Implication for research and clinical practice, page 21 - 22
PLAIN LANGUAGE SUMMARY			
Plain language summary	3	If allowed by the journal, provide a plain language summary with background information and key findings.	Abstract, results, conclusions, key words. Page 2
OPEN SCIENCE			
Registration and protocol	4a	Provide registration information for the review, including register name and registration number, or state that the review was not registered.	Methods, search

Section and Topic	#	Checklist item[a]	Location
			strategy, page 6 (paragraph 1)
	4b	Indicate where the review protocol can be accessed, or state that a protocol was not prepared.	Availability of data and materials, page 23
	4c	Describe and explain any amendments to information provided at registration or in the protocol.	N/A
Support	5	Describe sources of financial or non-financial support for the review, and the role of the funders in the review.	Funding, page 23
Competing interests	6	Declare any competing interests of review authors.	Competing interests, page 23
Availability of data, code, and other materials	7	Report which of the following are publicly available and where they can be found: template data collection forms; data extracted from included studies; data used for all analyses; analytic code; any other materials used in the review.	Availability of data and materials, page 23
INTRODUCTION			
Rationale	8	Describe the rationale for the review in the context of existing knowledge.	Introduction, page 5 (last paragraph) page 6 (paragraph 1)
Objectives	9	Provide an explicit statement of the objective(s) or question(s) the review addresses and include as applicable the following (in any order): outcome domain of interest, population of interest, name/type of OMIs of interest, and measurement properties of interest.	Aim, page 5 (paragraph 2)
METHODS			
Followed guidelines	10	Specify, with references, the methodology and/or guidelines used to conduct the systematic review.	Methods, Search

Section and Topic	#	Checklist item[a]	Location
			strategy, page 4(last paragraph), page 5 (paragraph 1)
Eligibility criteria	11	Specify the inclusion and exclusion criteria for the review.	Methods, study selection, page 6 (paragraphs 4, 5
Information sources	12	Specify all databases, registers, preprint servers, websites, organizations, reference lists and other sources searched or consulted to identify studies. Specify the date when each source was last searched or consulted.	Methods, search strategy, page 6 (paragraph 2, 3)
Search strategy	13	Present the full search strategies for all databases, registers, and websites, including any filters and limits used.	Methods, search strategy, page 6 (paragraph 2, 3)
Selection process	14	Specify the methods used to decide whether a study met the inclusion criteria of the review, e.g., including how many reviewers screened each record and each report retrieved, whether they worked independently, and if applicable, details of automation tools/AI used in the process.	Methods, data collection, page 7 (paragraphs 1, 2)
Data collection process	15	Specify the methods used to collect data from reports, e.g., including how many reviewers collected data from each report, whether they worked independently, any processes for obtaining or confirming data from study investigators, and if applicable, details of automation tools/AI used in the process.	Methods, data collection, page 7

Section and Topic	#	Checklist item[a]	Location
			(paragraphs 1, 2)
Data items	16	List and define which data were extracted (e.g., characteristics of study populations and OMIs, measurement properties' results, and aspects of feasibility and interpretability). Describe methods used to deal with any missing or unclear information.	Results, Characteristic of included assessment instruments, page 9 (Table 1)
Study risk of bias assessment	17	Specify the methods used to assess risk of bias in the included studies, e.g., including details of the tool(s) used, how many reviewers assessed each study and whether they worked independently, and if applicable, details of automation tools/AI used in the process.	Methods, data collection page 7 (paragraph 2) and Evaluation of Selected Studies, page 7 - 9 (paragraph 1)
Measurement properties	18	Specify the methods used to rate the results of a measurement property for each individual study and for the summarized or pooled results, e.g., including how many reviewers rated each study and whether they worked independently.	Results, characteristics of validation studies using cybersickness assessment instruments relates to immersive virtual reality, page 15 (table

Section and Topic	#	Checklist item[a]	Location
			2)
Synthesis methods	19a	Describe the processes used to decide which studies were eligible for each synthesis.	Methods, Study selection, page 6 (paragraphs 4 and 5)
	19b	Describe any methods used to synthesize results.	Results, COSMIN - based evaluation of measurement properties, page 15 (table 3)
	19c	If applicable, describe any methods used to explore possible causes of inconsistency among study results (e.g., subgroup analysis).	N/A
	19d	If applicable, describe any sensitivity analyses conducted to assess robustness of the synthesized results.	Results, COSMIN - based evaluation of measurement properties, page 15 (table 4)
Certainty assessment	20	Describe any methods used to assess certainty (or confidence) in the body of evidence.	Results, COSMIN - based evaluation of measurement

Section and Topic	#	Checklist item[a]	Location
			properties, page 16 (table 6)
Formulating recommendations	21	If appropriate, describe any methods used to formulate recommendations regarding the suitability of OMIs for a particular use.	N/A
RESULTS			
Study selection	22a	Describe the results of the search and selection process, from the number of records identified in the search to the number of study reports included in the review, ideally using a flow diagram. If applicable, also report the final number of OMIs included and the number of study reports relevant to each OMI. [T]	Results, Study selection, page 9 (paragraph 1, flow diagram in figure 1)
	22b	Cite study reports that might appear to meet the inclusion criteria, but which were excluded, and explain why they were excluded.	Results, Study selection, page 9 (paragraph 1, flow diagram in figure 1)
OMI characteristics	23a	Present characteristics of each included OMI, with appropriate references. [T]	Results, Characteristics of included assessment instruments, page 9 (paragraph 3) to page 14 (paragraph 1)
	23b	If applicable, present interpretability aspects for each included OMI. [T]	Results, Characteristics

Section and Topic	#	Checklist item[a]	Location
			of included assessment instruments, page 9 (table 1)
	23c	If applicable, present feasibility aspects for each included OMI. [T]	N/A
Study characteristics	24	Cite each included study report evaluating one or more measurement properties and present its characteristics. [T]	Results, Characteristics of validation studies using cybersickness assessment instruments related to immersive virtual reality, page 15 (table 2)
Risk of bias in studies	25	Present assessments of risk of bias for each included study. [T]	Results, COSMIN-based evaluation of measurement properties, page 15, (Table 4)
Results of individual studies	26	For all measurement properties, present for each study: (a) the reported result and (b) the rating against quality criteria, ideally using structured tables or plots. [T]	Results, COSMIN-based evaluation of measurement properties,

Section and Topic	#	Checklist item[a]	Location
			page 15(table 3 and 4)
Results of syntheses	27a	Present results of all syntheses conducted. For each measurement property of an OMI, present: (a) the summarized or pooled result and (b) the overall rating against quality criteria. [T]	Results, COSMIN-based evaluation of measurement properties, page 15(table 5 and figure 2)
	27b	If applicable, present results of all investigations of possible causes of inconsistency among study results.	N/A
	27c	If applicable, present results of all sensitivity analyses conducted to assess the robustness of the synthesized results.	N/A
Certainty of evidence	28	Present assessments of certainty (or confidence) in the body of evidence for each measurement property of an OMI assessed. [T]	Results, COSMIN-based evaluation of measurement properties, page 16 (table 6)
Recommendations	29	If appropriate, make recommendations for suitable OMIs for a particular use.	Results, COSMIN-based evaluation of measurement properties, page 16 (table 6)
DISCUSSION			

Section and Topic	#	Checklist item[a]	Location
Discussion	30a	Provide a general interpretation of the results in the context of other evidence.	Results, Clinical application contexts, page 16 - 17
	30b	Discuss any limitations of the evidence included in the review.	Discussion limitations, page 22 (paragraph 2)
	30c	Discuss any limitations of the review processes used.	N/A
	30d	Discuss implications of the results for practice, policy, and future research.	Results, Clinical application contexts, page 16 - 17

It is strongly recommended that this checklist is used in conjunction with the PRISMA-COSMIN for OMIs 2024 Explanation and Elaboration (E&E) document for important clarification on the checklist items. The PRISMA-COSMIN for OMIs 2024 statement checklist is distributed under the terms of the Creative Commons license.

[a] If an item is marked with [T], a template for data visualization is available. These templates can be downloaded from www.prisma-cosmin.ca.

[b] Item #2.1 in the PRISMA-COSMIN for OMIs 2024 Abstracts checklist refers to the title. Item #2.1 in the Abstracts checklist is identical to item #1 in the Full Report checklist.

From: Elsman EBM, Mokkink LB, Terwee CB, Beaton D, Gagnier JJ, Tricco AC, et al. Guideline for reporting systematic reviews of outcome measurement instruments (OMIs): PRISMA-COSMIN for OMIs 2024. *The Journal of Clinical Epidemiology* (2024), doi: https://doi.org/10.1016/j.jclinepi.2024.111422.
